# Divergent trends in functional and phylogenetic structure in reptile communities across Africa

**DOI:** 10.1038/s41467-018-07107-y

**Published:** 2018-11-08

**Authors:** Till Ramm, Juan L. Cantalapiedra, Philipp Wagner, Johannes Penner, Mark-Oliver Rödel, Johannes Müller

**Affiliations:** 1 Museum für Naturkunde, Leibniz Institute for Evolution and Biodiversity Science, Invalidenstraße 43, D-10115 Berlin, Germany; 2 School of BioSciences, University of Melbourne, Parkville, VIC 3010 Australia; 3 Museum Victoria, GPO Box 666, Melbourne, VIC 3001 Australia; 4 Dpto Ciencias de la Vida, Universidad de Alcalá, 28805 Alcalá de Henares, Madrid Spain; 5 Allwetterzoo Münster, Sentruper Str. 315, D-48161 Münster, Germany; 6 Department of Biology, Villanova University, 800 E. Lancaster Avenue, Villanova, 19085 PA USA; 7 University of Freiburg, Chair of Wildlife Ecology & Management, Tennenbacher Str. 4, D-79106 Freiburg, Germany; 8 Berlin-Brandenburg Institute of Advanced Biodiversity Research, Altensteinstr. 34, 14195 Berlin, Germany

## Abstract

Despite extensive research on ecological community compositions, general patterns across large-scale environmental gradients have remained unclear. A widely used explanatory model is the stress dominance hypothesis (SDH), predicting that the relative influence of environmental filtering is greater in stressful habitats while competition is more important in benign environments. Here, we test the SDH using African squamates as a model system to facilitate general predictions on community structures amidst changing global environments. For the first time we investigate changes in functional, phylogenetic and species diversity across continental, environmental gradients within a multidimensional, phylogenetically informed approach. Results suggest that phylogenetic patterns of African squamates were likely shaped by clade-specific biogeographic histories, whereas functional structure reflects SDH predictions. We further detected significant structuring at both local and regional spatial scales, emphasizing the impact of regional-historical processes on local assemblages, and the need for broad conceptual frameworks to detect general patterns of community composition.

## Abstract (web summary)

The biogeographic drivers of reptile diversity are poorly understood relative to other animal groups. Here, using a dataset of distributions of African squamates, the authors show that environmental filtering explains diversity in stressful habitats while competition explains diversity in benign habitats.

## Introduction

Despite an increasing need to assess relationships between environmental change and community responses^1^, general patterns of community compositions, especially along large-scale climatic gradients, remain poorly understood^2^. Two deterministic processes, environmental filtering and competition, are generally thought to play an important role in structuring ecological communities^3–5^. One proposition, the so-called stress dominance hypothesis (SDH), predicts that along a gradient of increasing environmental stress the relative proportion of environmental filtering increases while the importance of competition is higher in less stressful environments^6–8^. Testing the generality of the SDH is of significant importance, since information about the variation of assembly processes along environmental gradients facilitate general predictions on patterns of community compositions^9–11^. Yet, support for the SDH has been ambivalent so far^7,8,10,12–18^. Previous research largely focused on local communities, ignoring the impact of regional processes on local scale assemblages (but see Ricklefs^19,20^ and Cantalapiedra et al.^21^) and most studies do not consider geographic areas with extensive environmental gradients across larger geographic scales, which is mandatory for inferring the generality of the SDH^8^.

To disentangle the relative influence of ecological and biogeographical or evolutionary processes on various scales, it is crucial to carry out multidimensional analyses of community structures^2,11^. Trait-based functional diversity measures are probably more sensitive to reflect local ecological processes, while phylogenetic patterns in combination with species richness can provide information on processes acting at higher spatio-temporal levels, such as a clades biogeographic history^2,22^. In support of this view, several studies showed that phylogenetic diversity is probably a poor proxy for functional diversity and vice versa^23–27^ even when the traits under consideration show a phylogenetic signal^16,28,29^. Also, to increase the chances of detecting generalisable patterns, examinations at different spatial scales (i.e. local vs regional species pools) are needed. While ecologically driven processes are likely to be observed at smaller scales, larger scales should reflect long-term biogeographic or evolutionary processes^5,21,30,31^.

Here, we combine the use of multiple community assembly predictors (phylogenetic diversity, functional diversity, and species richness) with the evaluation of their respective changes across an extensive environmental gradient in a multiscale, continent-wide analysis of African squamate reptiles (lizards and snakes), one of the major living clades of vertebrates. Due to its variety in biomes and ecoregions ranging from extreme deserts to moist evergreen forests, Africa represents a perfectly suited area for studying the effects of environmental gradients on community structure^32^.

We expect environmental filtering to be more prevalent in arid deserts, leading to functionally similar species (low functional diversity) in local communities, while the effects of competition, resulting in character displacement or competitive exclusion (via limiting similarity), should be reflected in the occurrence of functionally dissimilar species (high functional diversity) in local assemblages in more humid regions^6^. However, if traits are linked with species fitness (and if these traits show a phylogenetic signal), competitive exclusion may instead result in phylogenetic clustering^33^. By examining different spatial scales (i.e. local communities and biomes), we test if different assembly processes shape local and regional species pools (see e.g. Pavoine & Bonsall^2^; Cantalapiedra et al.^21^), and discuss our findings by incorporating phylogenetic diversity, species richness and the biogeographic history of African squamates.

We investigate the phylogenetic and functional structure of squamate local assemblages (tested against both continental and biome species pools), the pattern of such structuring across different biomes, as well as the correlations of community structure parameters with climatic variables. Our results show that the functional community structure of African squamate reptiles largely follows the predictions of the SDH. Instead, the phylogenetic diversity shows a very different pattern, which is most likely driven by the different biogeographic histories of African lizards and snakes. Significant structuring of large-scale (biome) assemblages is consistent with local community patterns and highlights the importance of assembly processes acting at large spatio-temporal levels.

## Results

### NRI vs NTI

The net relatedness index^3^ (NRI) as well as the nearest taxon index^3^ (NTI) were significantly positively correlated (Pearson correlations, *p* < 0.025; Supplementary Data 3) with each other, both for phylogenetic and functional distances. However, we found no clear geographic pattern in NTI neither regarding the number of significantly structured communities or the correlations with environmental variables. Whereas NTI is especially powerful for detecting the clumping of clustering at the tips of phylogenies, (i.e. if terminal taxa are clustered or overdispersed^3^), it is especially likely to detect clustering in species assemblages of larger geographic extent, probably reflecting more recent radiations within these regions^34^. In our study, assemblages represent larger geographic areas, such as national parks or entire biomes, which are likely to comprise a variety of species from different, larger clades. In the following, therefore we will focus on the NRI results, while differences to NTI will be discussed when appropriate.

### Phylogenetic structure

**Fig. 1:**
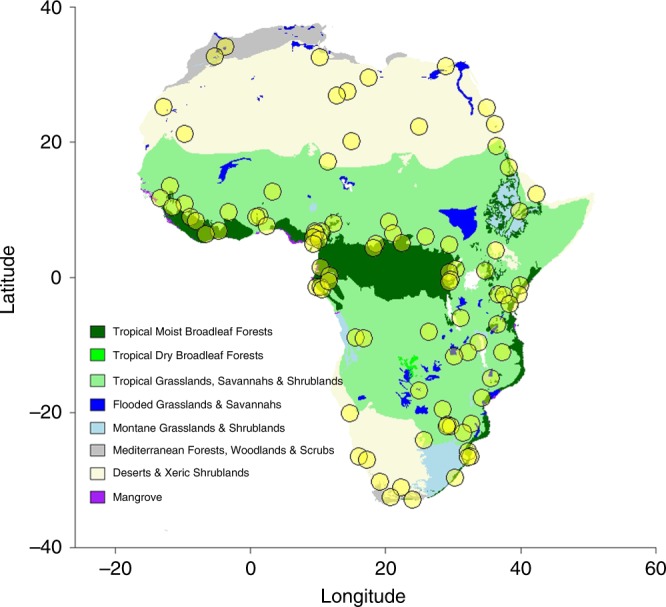
Distribution of the localities used in the analyses

**Fig. 2:**
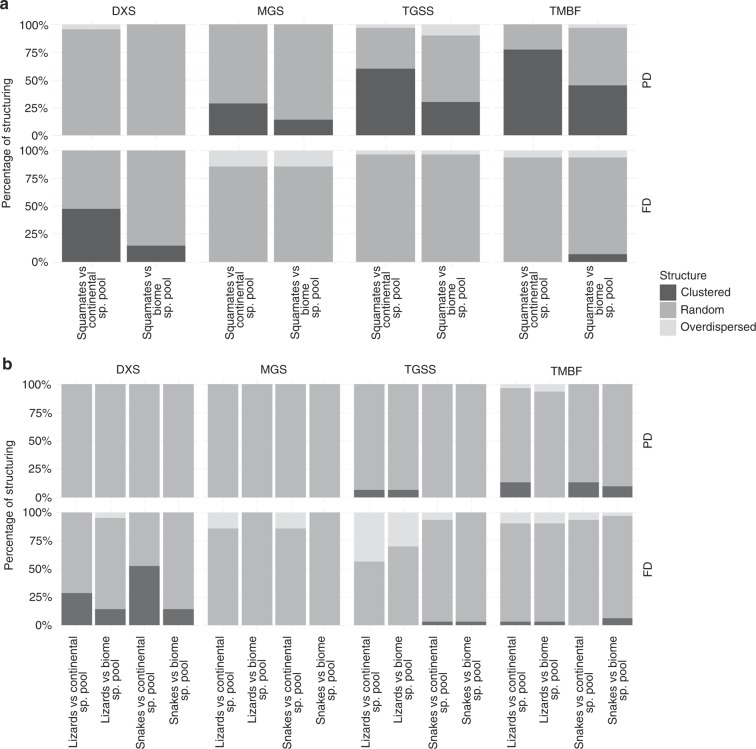
Phylogenetic and functional diversity in different biomes. Proportions of significantly clustered, overdispersed and randomly structured local communities (NRI) in different biomes (95% CI), according to phylogenetic (PD) and functional (FD) diversity, tested against the continental and the biome species pool. **a** Complete data set of all African squamates. **b** Comparison of lizards and snakes. DXS deserts and xeric shrublands (*n* = 21), MGS montane grasslands and shrublands (*n* = 7), TGSS tropical grasslands, savannahs, and shrublands (*n* = 30), TMBF tropical moist broadleaf forests (*n* = 31)

**Fig. 3:**
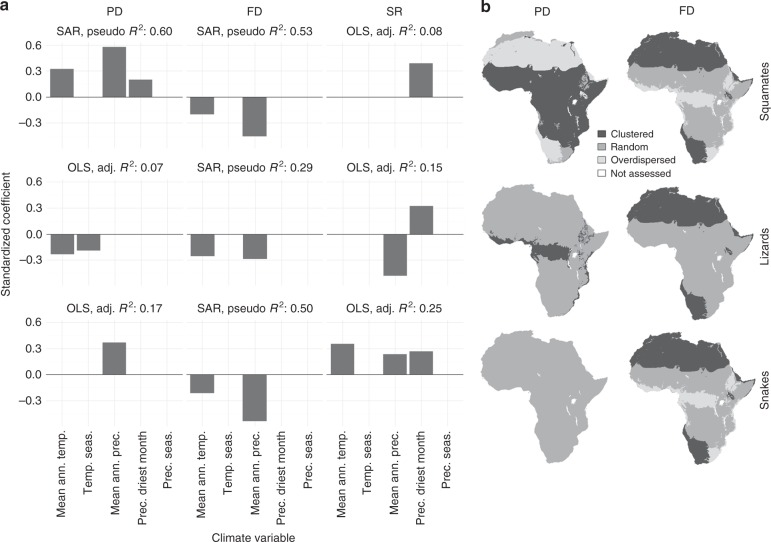
Climate model coefficients and structuring of regional assemblages. **a** Standardised correlation coefficients of significant predictors of phylogenetic (PD) and functional (FD) diversity as well as species richness (SR) at local scale, derived from the best respective ordinary least squares (OLS) or spatial autoregressive (SAR) model. **b** Phylogenetic and functional structuring of biome assemblages, tested against the continental species pool (95% CI). adj. *R*² adjusted *R*², pseudo *R*² Nagelkerke pseudo *R*², mean ann. temp. mean annual temperature, temp. seas. temperature seasonality, mean ann. prec. mean annual precipitation, prec. driest month precipitation of the driest month, prec. seas. precipitation seasonality. To increase normality, temperature seasonality (Bio4) was log transformed, precipitation of the driest month (Bio14) was log(x + 1) transformed and precipitation seasonality (Bio15) was square root transformed

**Fig. 4:**
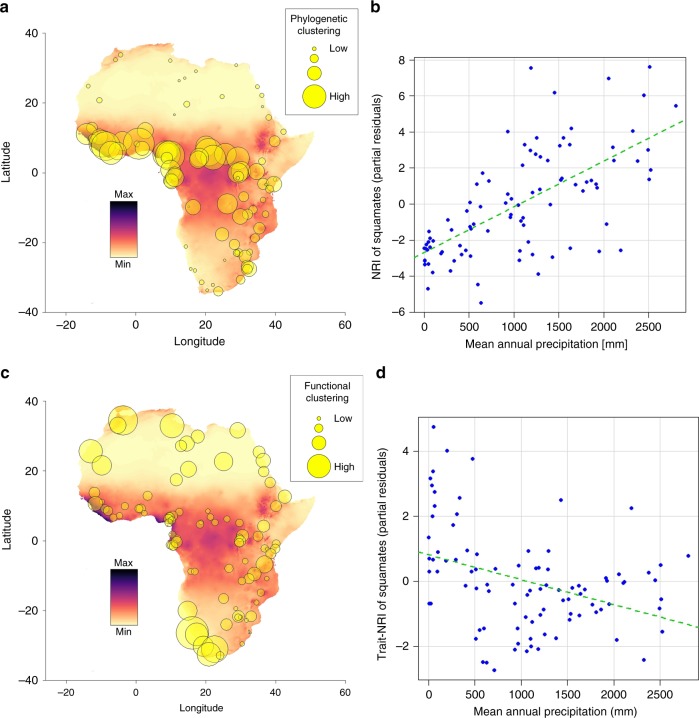
Spatial distribution of phylogenetic and functional structure. **a**, **c** Spatial distribution of phylogenetic and functional structure of African squamates, showing correlations with mean annual precipitation (most important variable according to AICc weights). The colour gradient represents a gradient of mean annual precipitation from high (dark) to low (light). **b**, **d** Partial residual plots of phylogenetic and functional structure and mean annual precipitation of the best respective ordinary least squares models

Of the 92 evaluated squamate communities (Fig. 1), 45 (48.9%) were significantly clustered, whereas 2 (2.2%) were significantly overdispersed when null models were created using the continental species pool. Significantly clustered communities occurred in the humid biomes (Flooded Grasslands & Savannahs, Tropical Grasslands, Savannahs & Shrublands, Tropical Moist Broadleaf Forests), as well as in Montane Grasslands & Shrublands, whereas no clustering was present in the more arid biomes (Deserts & Xeric Shrublands, Mediterranean Forests, Woodlands & Scrubs) (Fig. 2a). The significantly overdispersed communities occurred in the Tropical Grasslands, Savannahs & Shrublands biome, as well as in Deserts & Xeric Shrublands (Fig. 2a). Less significant structuring was observed when communities were tested against their respective biome species pool (24 out of 92 communities were significantly clustered and 4 were significantly overdispersed), while significant clustering was still present only in the humid biomes (Fig. 2a). Subsets leaving out faunal lists for entire countries, species with non-discrete lifestyle trait states, fossorial species or manually added species all showed a very similar pattern when compared to the complete data set, indicating that our data are robust towards methodological biases (Supplementary Data 3‒6, Supplementary Figures 4‒7). NRI showed positive correlations with mean annual temperature (Bio1), mean annual precipitation (Bio12) and precipitation of the driest month (Bio14), indicating higher NRI values (more clustering) in humid regions (Figs. 3a and 4). At the spatial biome scale, the three humid biomes constituted significantly clustered subsets of the African species pool, whereas the Deserts & Xeric Shrublands biome showed significant overdispersion (Fig. 3b).

Lizards showed less phylogenetic structuring, with 7 (7.6%) significantly clustered and 1 (1.1%) overdispersed community when tested against the continental species pool. Clustering was present in arid as well as tropical biomes, with no apparent spatial structure (Fig. 2b). The number of significantly structured communities lowered when the NRI of localities was calculated using biome species pools (2 clustered and 2 overdispersed communities; Fig. 2b). The correlation of the NRI of lizards with climatic variables was ambiguous (negative correlation with mean annual temperature, but also negative correlation with temperature seasonality) (Fig. 3a). However, on the spatial scale of biomes, the Tropical Moist Broadleaf Forests biome was significantly clustered (Fig. 3b).

Snakes showed significant clustering only in the Tropical Moist Broadleaf Forests biome (4 out of 92 communities when compared to continental and 3 out of 92 when compared to the biome species pool) (Fig. 2b). NRI was positively correlated with mean annual precipitation (Fig. 3a). On the biome scale no significant NRI structuring was present. (Fig. 3b).

### Trait structure

The trait-NRI of the combination of body size and lifestyle revealed 12.0% (11 out of 92) of the communities to be significantly clustered and 4.3% (4 out of 92) to be overdispersed when compared to the continental species pool. All clustered communities were located in the arid biomes (47.6% of communities within Deserts & Xeric Shrublands were clustered) whereas the overdispersed communities were spread across the remaining biomes (Fig. 2a). When tested against the biome species pool, five communities were significantly clustered while four were overdispersed (Fig. 2a). According to the best respective SAR model (pseudo *R*^2^ = 0.53), the localities trait-NRI was further negatively correlated with mean annual temperature and mean annual precipitation, indicating a higher amount of trait clustering in arid regions (Figs. 3a and 4). At the biome scale this pattern remained consistent, with the two arid biomes being significantly clustered, while the Tropical Moist Broadleaf Forests and the Montane Grasslands & Shrublands biomes were significantly overdispersed (Fig. 3b). A similar NRI pattern was observed in the phylogenetic subsets of lizards and snakes. Nearly all significantly clustered localities were present in the arid biomes (8 out of 9 for lizards and 12 out of 13 for snakes when compared to continental species pool), while overdispersed localities were only found in humid biomes and Montane Grasslands & Shrublands (Fig. 2b). Interestingly, 17 lizard and 5 snake communities were significantly overdispersed, all of which were distributed in the humid biomes. When tested against the biome species pools the number of significantly structured communities was relatively lower (5 clustered and 13 overdispersed for lizards and 6 clustered and 1 overdispersed for snakes; Fig. 2b). The trait-NRI at the locality scale of both groups was negatively correlated with mean annual precipitation (Bio12) and mean annual temperature (Bio1; Fig. 3a). At the biome scale the two arid biomes were significantly clustered (Fig. 3b) for both groups. In addition, the Tropical Moist Broadleaf Forests biome and the Montane Grasslands & Shrublands biome was significantly overdispersed for snakes (Fig. 3b).

The community mean of log10 (body size) was significantly positively correlated with mean annual temperature (Bio1) and mean annual precipitation (Bio12; see Supplementary Figure 1).

### Species richness

**Fig. 5:**
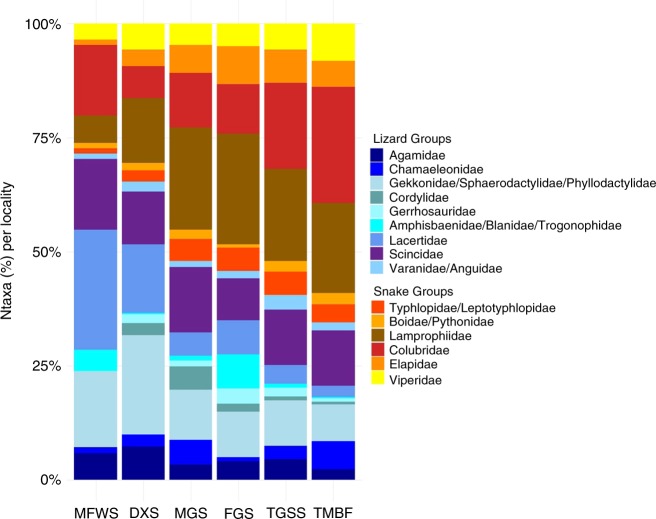
Species richness of African squamates in different biomes. Relative number of taxa per locality of different squamate subgroups in different biomes. MFWS Mediterranean forests, woodlands and scrubs (*n* = 2), DXS deserts and xeric shrublands (*n* = 21), MGS montane grasslands and shrublands (*n* = 7), FGS flooded grasslands and savannahs (*n* = 1), TGSS tropical grasslands, savannahs and shrublands (*n* = 30), TMBF tropical moist broadleaf forests (*n* = 31)

The species richness of African squamates showed significantly positive correlations with precipitation of the driest month (Bio14), indicating more species in humid environments, but the explained variance of the model was very low (8%; Fig. 3a). A post hoc test, comparing the means of species richness per locality in different biomes, revealed no significant differences for all African squamates (see Supplementary Figure 3). The species richness of lizards was instead negatively correlated with mean annual precipitation (Bio12) and positively correlated with precipitation of the driest month (Bio14; Fig. 3a). However, a post hoc test of the means of species richness per locality in different biomes indicated a significantly higher number of species in Deserts & Xeric Shrublands, compared to Tropical Moist Broadleaf Forests (see Supplementary Figure 3). Snakes showed an opposing richness gradient compared to lizards (Fig. 5). Snake species richness was positively correlated with mean annual temperature (Bio1), mean annual precipitation (Bio12) and precipitation of the driest month (Bio14), indicating a higher species richness in humid regions (Fig. 3a). Also, the post hoc test revealed a significantly higher number of species in Tropical Moist Broadleaf Forests and Tropical Grasslands Savannahs & Shrubs when compared to Deserts & Xeric Shrublands (see Supplementary Figure 3).

## Discussion

Our results show that the phylogenetic community structure of African squamates and the combination of two traits, body size and lifestyle, which are hypothesised to play a major role in competitive interactions, show completely opposing patterns along an extensive aridity gradient both at the community and biome scale. The functional structure of body size and lifestyle of African squamates largely confirms what would be predicted by the SDH. However, we observed the highest number of significantly structured localities when compared to continental species pools, and further found significant structuring also at larger spatial scales (biome assemblages). In contrast, the number of significantly structured localities lowered when tested against their respective biome species pools. This indicates that macroecological processes, which likely were responsible for shaping regional species pools, in turn influence the structure of localities^21,31^. Thus, the observed pattern in both phylogenetic and functional structure emphasizes the influence of regional and historical processes on local community assembly. To our knowledge, these findings represent the first evidence of a fundamental change of assembly mechanisms along a continent-wide environmental gradient.

In arid environments, a low functional diversity coupled with a high-relative phylogenetic diversity most likely reflects habitat filtering of convergent ecomorphologies, at least with respect to the two traits investigated in our study. These interpretations support the assumption of the SDH that in stressful environments habitat filtering affects community structure more strongly than competition^6–8^. However, since significant functional clustering was observed also at the biome level, environmental filtering within arid regions probably has acted over larger spatio-temporal scales in African squamates. This view is also supported by the relatively lower number of functionally structured localities within Deserts & Xeric Shrublands, when compared to their respective biome species pool, indicating that, within biome, differences of localities do not represent intense environmental filters. Strong evidence for the occurrence of habitat filtering in regional communities suggests that the geographically distinct Sahara and Namib deserts constitute significantly clustered functional subsets of the investigated species pool, even when considered together as a single biome.

Because a high-relative phylogenetic diversity was observed in arid regions in both communities and biomes, the trait combinations of their respective members are most likely convergent^2^. The SDH predicts that environmental filtering acts on traits promoting stress tolerance, thus leading to a high degree of convergence^8^. With respect to our study group, this would imply that disparate lineages evolved similar trait combinations despite having evolved in (or dispersed into) Africa at very different times. Within African squamates, there is indeed some evidence that several clades entered the African arid biomes independently during different periods of the Cenozoic: Lacertids, e.g. originated most likely in Eurasia and entered Africa sometime in the late Paleogene^35,36^, followed by several Neogene radiations within Africa. Also for agamids^37,38^ and gekkotans^39,40^ independent invasions into the arid African biomes have been suggested. These independent invasions of phylogenetically distinct groups are probably reflected by a high-relative phylogenetic diversity in arid regions, as observed in the NRI pattern. In contrast, there were some significantly clustered localities in arid regions according to NTI, probably representing more recent speciation events within clades that are well spread across the entire phylogeny.

The environmental conditions in this biome seem to favour smaller body sizes in combination with a terrestrial lifestyle (see Supplementary Figures 1, 2). Small body size may be a consequence of the specific thermoregulatory requirements^41^ of ectothermic squamates, whose body temperatures are largely dependent on ambient temperatures. Our model examining the change of mean body size values of localities along an environmental gradient suggests that African squamates reach larger body sizes in more humid regions (see Supplementary Figure 1, Supplementary Data 5). Similar conditions were proposed for all squamates by Ashton & Feldman^41^, who stated that the smaller body sizes of squamates in more seasonal habitats may be driven by the selection of an increased surface to volume ratio, which possibly allows for a more precise behavioural thermoregulation compared to larger body sizes with lower surface to volume ratios. This hypothesis may hold true since a more precise thermoregulation would be advantageous in environments with increased thermal stress, thus favouring smaller size.

On the humid end of the gradient, a low-relative phylogenetic diversity in conjunction with a high-relative functional diversity indicates ecological sorting within a group of closely related taxa, possibly as a consequence of increased competition in more benign environments^6–8^ as proposed by the SDH. Consistent with other studies^16,30,42^, we found greater trait divergence in local (and regional) assemblages consisting of phylogenetically closely related species. Such a pattern is frequently associated with biotic interactions^2,30^^,^, whereas other mechanisms, such as facilitation between species of divergent trait states, may also be considered^30^. However, since significant trait overdispersion is present also at the biome scale, it seems unlikely that this pattern is solely driven by present-day local ecological factors. Instead, our results seem consistent with a proposition of the Evolutionary Interaction Hypothesis^30^, stating that the evolutionary divergence of traits among species, mediated by interactions, occurs on relatively large temporal scales, and thus is probably reflected across all localities of similar habitats rather than in single local communities. Indeed, significant trait overdispersion was present across biomes, while only in a few local communities significant overdispersion was detected. Thus, this pattern emphasizes the influence of macroevolutionary patterns on the present-day diversity of local species assemblages^19–21^, with past biotic interactions still being the driving force. However, the strong phylogenetic clustering in humid African regions, which is present both in communities and in biomes, seems to be a consequence of the high species richness of snakes, which form a monophyletic group inside paraphyletic lizards^43–45^. Further evidence for this assumption arises from the lower amount of phylogenetic structuring at smaller phylogenetic levels (lizards & snakes) at both biome and locality scales.

While the amount of significant phylogenetic structuring is relatively low in both lizards and snakes, the SDH still seems to be reflected in the trait combination of body size and lifestyle, also at smaller phylogenetic levels. No differences were observed regarding the functional pattern of both groups in arid regions. Similar to the pattern observed for all African squamates, both subgroups show a low-relative functional diversity at local scales and significant functional clustering in biomes. However, differences were detected at the spatial scale of functional overdispersion in humid African regions as well as in the patterns of species richness, which seem to be related to the different biogeographic histories of African lizards and snakes.

In snakes, the trait combination of body size and lifestyle follows the predictions of the SDH at both community and biome levels, whereas the phylogenetic diversity showed no significant structuring in biomes (i.e. there were only a few clustered localities in the humid biomes). As was shown before^46^, African snakes follow a classical latitudinal diversity gradient, with more species being present at lower latitudes. This richness gradient might be shaped by tropical niche conservatism^47,48^, which was already suggested to be responsible for the high species richness of alethinophidian snakes in humid African regions^49^. Such a scenario would fit the observed pattern of significant functional overdispersion for African snakes on a regional scale for the Tropical Moist Broadleaf Forests biome. If snakes indeed show a pattern of tropical niche conservatism in humid African regions, past interactions could likely have mediated evolutionary trait divergence within these regions over millions of years^30^.

Lizards, on the other hand, do not show significant functional overdispersion across biomes, but still display a higher relative functional diversity in humid regions at the locality level (including a high number of significantly overdispersed communities in the Tropical Grasslands, Savannahs & Shrublands biome). In contrast to snakes, the Tropical Moist Broadleaf Forests biome contained a significantly clustered phylogenetic subset of lizard species. Considering also the lower species richness of lizards in this area, this finding may imply that this biome was colonised rather recently and only by a subset of the different lizard clades^2,47^. If correct, biotic interactions might have mediated trait divergence in lizards within this biome only over a shorter period of time, in contrast to snakes. Thus, the differences regarding the spatial scales of significant functional clustering could reflect differences in the period of time over which assembly processes occur^5,21,31^. However, the lack of significant functional overdispersion at the biome scale might also be a consequence of the lower range sizes of lizards compared to snakes^50^.

When lizards were analysed separately, a tendency for higher species richness was observed in arid regions (see also Lewin et al.^46^). Since the amount of functional clustering was still high, it seems unlikely that biotic interactions mediated speciation within these regions. For several clades of African squamates, the aridification that took place during the Miocene, especially in North African regions, is thought to be responsible for e.g. vicariance induced speciation events^51–54^, which might explain the distinctive richness gradient in African lizards. However, it might also be possible that the spatial scale of the present study is not sufficient for reflecting effects of species interactions at finer spatial resolutions, which, for example, were shown to be important for structuring desert lizard communities in Australia^55^. Another possibility may be that additional traits beyond body size and lifestyle mediate the outcome of competitive interactions, but such an assessment is beyond the scope of the present study.

Our study provides support for the SDH regarding the functional structure of African squamates on a continent-wide scale. While environmental conditions most likely constrain the functional diversity in arid environments, the high functional diversity in humid African regions might reflect a greater relative importance of biotic interactions. The observed structuring at regional levels and the lower structuring of localities when tested against their biome species pools also suggest that both regional and historical processes may be relevant for the assembly of present-day local scale communities, and emphasize the importance of considering different spatial scales in studies of community assembly. In agreement with previous studies, phylogenetic diversity alone does not seem to be a useful proxy for functional diversity and rather appears to reflect different biogeographic histories, especially when considered in combination with species richness patterns. Our results suggest that the application of multidimensional analyses as well as the consideration of macroecological processes is crucial for detecting general patterns of community assembly.

## Methods

### Community composition and phylogeny

We compiled a species composition database of local communities of African squamates from the literature, complemented by own unpublished records (see Supplementary References). If not stated in the publications, georeferenced locality points were taken from the approximate centre of each locality. If necessary, taxon names were updated following The Reptile Database^56^ (a table containing synonyms can be found in Supplementary Data 1). After evaluating completeness and sampling efforts, based on expert experiences, we included 92 different localities from all over Africa and a total of 904 species (Fig. 1).

The large-scale phylogenetic tree of Pyron et al.^44^, containing 4161 squamate species, provided the basis for the phylogeny used in this study. The original tree by Pyron et al. was calibrated with the program treePL^57^, which uses a penalised likelihood method. This method is suitable for large phylogenies^57^, and outperforms clock models and nonparametric rate smoothing models by adding a roughness penalty, which increases as rates vary more rapidly across the tree^58^. We selected 14 calibration points following Jones et al.^59^, Head^60^ and Head et al.^61^ (see Supplementary Table 1). Of the included species, 386 (199 lizards and 187 snakes; see Supplementary Data 2) were not present in the phylogeny of Pyron et al., and had to be grafted onto the tree using the program TreeGraph2^62^. For several clades, independently published phylogenies were used to insert the respective species at a reasonable position (see Supplementary References). Since the exact divergence ages were unknown, new branches were subsequently inserted at the mid-level of each target branch. If no separate phylogeny was present, species were inserted at the base of the respective genus or clade, thus creating polytomies. After calibration, the tree was pruned to the species present in our data set using the R package picante^63^ (Supplementary Data 8).

To test if species that were inserted manually into the phylogeny would affect the outcome of NRI or NTI we repeated our analyses including only those species that were present in the original tree of Pyron et al. (*n* = 518). As fossorial species’ record probabilities may be biased due to sampling intensity, we further repeated the analysis excluding all Amphisbaenidae, Trogonophidae, Blanidae, Typhlopidae, and Leptotyphlopidae (remaining *n* = 829). Since ten publications contained lists for whole countries, instead of more geographically restricted areas, a subset without those localities was created to preclude inaccuracies due to extensive size differences of the localities (remaining *n* = 818). Furthermore, we analysed the complete data set (*n* = 904), as well as lizards (*n* = 522) and snakes (*n* = 382) separately. For the analyses of smaller subsets, the tree was always pruned accordingly.

### Trait data

A combination of body size and lifestyle was chosen to define the functional groupings for the present study, because we hypothesise that squamates with a similar body size and a similar lifestyle compete more strongly with each other than taxa of different sizes and ecologies. In addition, data on lifestyle and body size are among the best sampled traits across Squamata, and especially body size is often used as a proxy for functional diversity^64,65^. However, we acknowledge that depending on size and lifestyle, squamate species might also compete with non-squamate taxa such as mammals or birds, potentially influencing patterns within squamates. Trait data for maximum body mass was obtained from Feldman et al.^66^, who used clade-specific allometric equations to convert measures of maximum body length of 9805 lepidosaur species to body mass in grams. The information on the maximum body length of *Saurodactylus brosseti*, the only species not present in the data set of Feldman et al., was obtained from Schleich et al.^67^ and converted to mass using the equation of Novosolov et al.^68^ for Sphaerodactylidae. For all subsequent analyses body mass values were log10 transformed to increase normality (tested using a Shapiro–Wilk test of normality). The information on lifestyle was obtained from a variety of published references, as well as expert opinions from experienced field herpetologists (see Supplementary Data 7 & Supplementary References). We assigned species to ten categories to represent the functional groupings, based on the species main zone of activity. These included fossorial, terrestrial, saxicolous, arboreal, generalist as well as intermediate states between terrestrial/arboreal, terrestrial/saxicolous, terrestrial/fossorial, saxicolous/arboreal and terrestrial/aquatic, which were ruled as distinct categories. Information on lifestyle could be obtained for 884 out of the 904 squamate species. However, it may well be possible that species which are intermediate between two discrete traits interact more strongly with species showing one of these two traits; for example, a terrestrial/saxicolous species is more likely to interact with either a terrestrial or a saxicolous species than with an arboreal form. Thus, to test how intermediate lifestyle trait states influence overall results, we repeated our analysis including only those species with discrete lifestyle traits (terrestrial, arboreal, fossorial, saxicolous, generalist; remaining *n* = 686).

### Environmental variables

To test for correlations between community structure parameters and climate, all 19 bioclim variables^69^ were extracted for each locality midpoint in R (grid cell resolution: 2.5 arc minutes, extraction method: bilinear; Supplementary Data 3), using the packages rgeos^70^ and raster^71^. Afterwards, the values were tested for correlation using the R package corrplot^72^ to sort out strongly correlated values, i.e. with a correlation higher than 0.75. Based on this test, the following variables were chosen: Bio1 (Annual mean temperature), Bio4 (Temperature seasonality, i.e. standard deviation*100), Bio12 (Annual precipitation), Bio14 (Precipitation of the driest month) and Bio15 (Precipitation seasonality). Each locality was assigned to a biome (Fig. 1, Supplementary Data 3) using the TEOW layer file^73^, as well as the R packages rgeos and raster.

### Community, phylogenetic and functional structures

We assessed the phylogenetic structure of African squamates using the NRI and NTI indexes for communities as well as for biomes. In order to identify scale-dependent assembly processes, we calculated communities' NRI and NTI using (1) the continental species pool and (2) their respective biome species pool to generate the null models (see e.g. Cardillo^31^, Cantalapiedra et al.^21^). For the second approach (locality versus biome species pool), Flooded Grasslands and Mediterranean biomes were excluded because they contained only one and two localities, respectively. The NRI and NTI values for biome assemblages were calculated using the continental species pool to generate the null models. The significance of the observed NRI and NTI is calculated by comparing the observed phylogenetic distance within a given locality with those from 5000 null values generated by shuffling taxon labels across the reference phylogenetic tree. These analyses were run using the R package picante^64^.

For the functional structure (i.e. the combination of body size and lifestyle), we computed pairwise distances among taxa based on their continuous and discrete metrics using a Gower distance matrix^74^. This distance matrix was used to assess the functional NRI and NTI of species assemblages using the same null model approach described above for phylogenetic distances. The NRI is equivalent to −1 * the standardised effect size of mean pairwise distance (MPD), and NTI id the equivalent of −1* the standardised effect size of the mean nearest neighbour distance (MNND). Especially MPD has been shown to represent a meaningful measure of functional diversity^8,66^. Furthermore, MPD is independent of species richness and robust to imbalanced phylogenies^75^. Functional NRI and NTI were calculated for all communities and biomes, respectively. *p*-values < 0.025 were considered as significantly clustered, whereas values > 0.975 indicated significant overdispersion. Community means of body size were calculated separately, to investigate possible spatial changes of body size.

### Statistical analyses

In order to evaluate the relative importance of the climate variables in explaining local phylogenetic structure, species richness, and functional structure of African squamates, ordinary least squares (OLS) models were performed, using the phylogenetic and functional metrics as well as species richness as dependent variable and the bioclim data as explanatory variables. A stepwise regression depending on AICc values was applied using the dredge function of the R package MuMin^76^. MuMin also provided the sum of AICc weights of predictors throughout all generated models (Supplementary Data 6), which represents the relative importance of each prediction variable across all created models during the stepwise regression. To increase normality, temperature seasonality (Bio4) was log transformed, precipitation of the driest month (Bio14) was log(*x* + 1) transformed and precipitation seasonality (Bio15) was square root transformed. To avoid inaccuracies due to spatial autocorrelation (e.g. Vamosi et al.^77^) we determined Moran's *I* values using four different row standardised spatial weight matrices (calculated with either the two, six or ten nearest neighbours, as well as a distance based weight matrix using a great circle distance of 1500 km). Moran's *I* was calculated by permutation tests (*n* = 999 permutations) to test for the presence of spatial autocorrelation in the model residuals. The highest number of significant Moran's *I* values (*p*-values < 0.05) throughout all created OLS models was observed, when calculated with a weight matrix of the six nearest neighbours (Supplementary Data 5). Because Moran's *I* values were significant for some of the OLS models, we implemented simultaneous autoregressive (SAR) models of the error type (following Kissling & Carl^78^) using the R package spdep^79^. For each OLS, we created four different SAR models, using the four types of weight matrices as described above, to test how the choice of weight matrices influences overall results. Moran's *I* values were then calculated for the residuals of the SAR models using the respective weight matrix. All SAR models were able to account for spatial autocorrelation, without major changes of the results (see Supplementary Data 5, 6). In cases where SAR models outperformed OLS models, based on AICc, or if spatial autocorrelation was present in the OLS residuals, we present the results of the SAR models created with a weight matrix calculated with the six nearest neighbours. Model performance was evaluated using adjusted *R*^2^ values for OLS models and Nagelkerke's pseudo *R*^2^ values for SAR models^80^.

Differences between the means of species richness of communities between different biomes were evaluated using either Tukey or Games–Howell post hoc tests, depending on the homogeneity of variances, which was tested using Fligner–Killeen tests. Mediterranean and flooded grasslands biomes were excluded from the post hoc tests, because they contained only two, and one localities, respectively.

## Electronic supplementary material

Description of Additional Supplementary Files

Supplementary Information

Supplementary Data 1

Supplementary Data 2

Supplementary Data 3

Supplementary Data 4

Supplementary Data 5

Supplementary Data 6

Supplementary Data 7

Supplementary Data 8

## Data Availability

All data generated or analysed during this study are included in this article (and its Supplementary Information files).
