# Supplementary material for: Divergent trends in functional and phylogenetic structure in reptile communities across Africa
Source: Nat Commun. 2018 Nov 8;9:4697. doi: 10.1038/s41467-018-07107-y (PMC6224532; doi:10.1038/s41467-018-07107-y)
Supplement: Supplementary file 1 — Description of Additional Supplementary Files [file 41467_2018_7107_MOESM1_ESM.docx]

**Supplementary Data 1**

Presence / absence matrix of African squamate occurrences in different localities with updated taxonomy.

**Supplementary Data 2**

List of species that were added manually to the tree of Pyron et al.

**Supplementary Data 3**

Locality data and results of community structure parameters of different localities and different data subsets

**Supplementary Data 4**

Results of community structure parameters of different biomes and different data subsets

**Supplementary Data 5**

Results of OLS and SAR models as well as Moran’s I values using different spatial weight matrices

**Supplementary Data 6**

Sum of Akaike weights of the different explanatory variables of all created models

**Supplementary Data 7**

Trait data for the African squamate species used in this study

**Supplementary Data 8**

Time calibrated tree of African squamates
