# Supplementary material for: Divergent trends in functional and phylogenetic structure in reptile communities across Africa
Source: Nat Commun. 2018 Nov 8;9:4697. doi: 10.1038/s41467-018-07107-y (PMC6224532; doi:10.1038/s41467-018-07107-y)
Supplement: Supplementary file 2 — Supplementary Information [file 41467_2018_7107_MOESM2_ESM.pdf]

Supplementary Information

Divergent trends in functional and phylogenetic structure in reptile communities across Africa

Ramm et al.

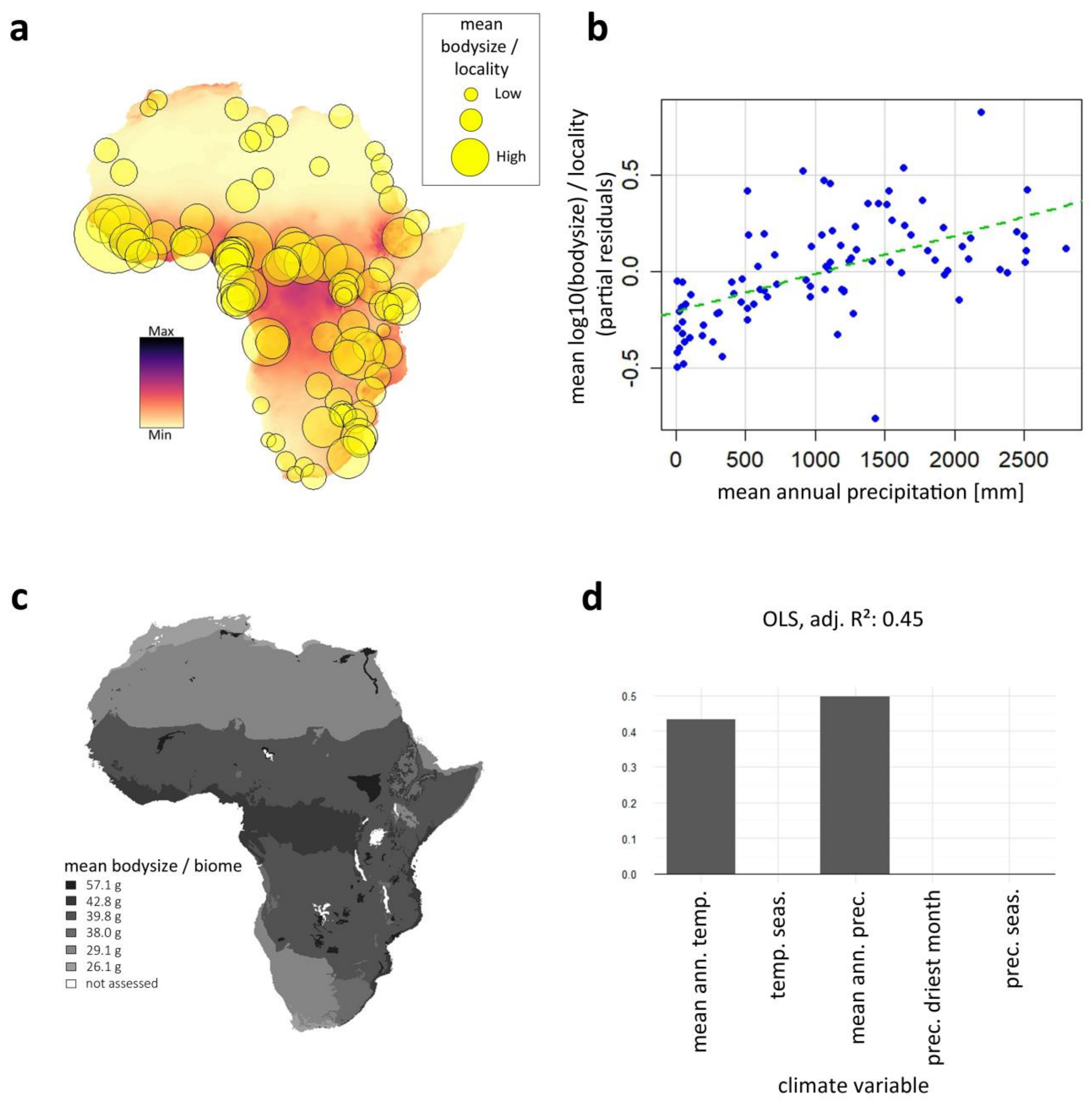

Supplementary Figure 1. Bodysize distribution of African squamates. a Spatial distribution of mean bodysize / locality of African squamates, showing correlations with mean annual precipitation (most important variable according to AICc weights). The color gradient represents a gradient of mean annual precipitation from high (dark) to low (light). **b** Partial residual plot of mean log(bodysize) / locality and mean annual precipitation of the best respective model. **c** Map showing the mean bodysize / biome of African squamates. **d** Standardized correlation coefficients of significant predictors of mean log(bodysize) / locality at local scale, derived from the best respective ordinary least squares (OLS) model. Abbreviations: adj. R^2 (adjusted R^2), mean ann. temp. (mean annual temperature), temp. seas. (temperature seasonality), mean ann. prec. (mean annual precipitation), prec. driest month (precipitation of the driest month), prec. seas. (precipitation seasonality).

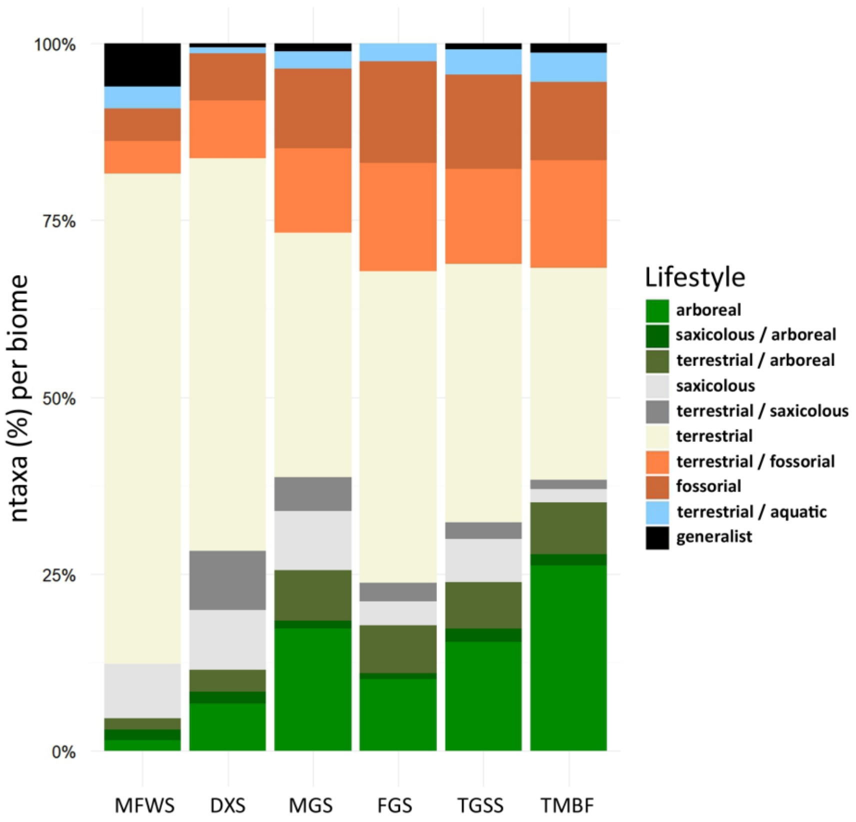

Supplementary Figure 2. Distribution of lifestyles of African squamates in different biomes. Relative amount of taxa of African squamates with different lifestyles in different biomes. Abbreviations: MFWS (Mediterranean forests, woodlands & scrubs; n = 2), DXS (deserts & xeric shrublands; n = 21), MGS (montane grasslands & shrublands; n = 7), FGS (flooded grasslands & savannahs; n = 1), TGSS (tropical grasslands, savannahs & shrublands; n = 30), TMBF (tropical moist broadleaf forests; n = 31).

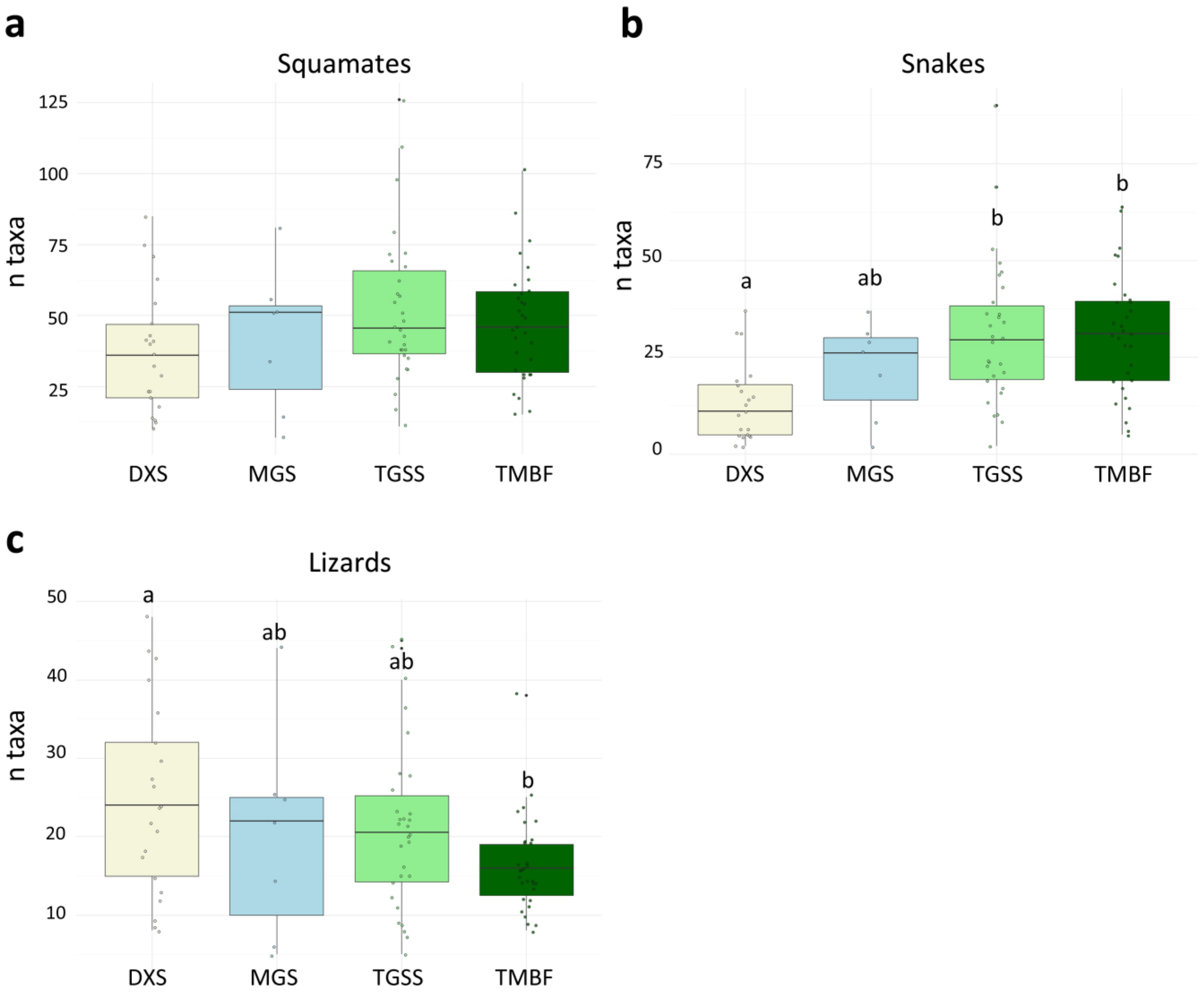

Supplementary Figure 3. Species richness of African squamates in different biomes. Boxplots of species richness of **a** squamates, **b** snakes and **c** lizards in different biomes. Different letters indicate significant differences according to tukey and games-howell posthoc tests (P-value < 0.05). Abbreviations: DXS (deserts & xeric shrublands; n = 21), MGS (montane grasslands & shrublands; n = 7), TGSS (tropical grasslands, savannahs & shrublands; n = 30), TMBF (tropical moist broadleaf forests; n = 31). Center line, median; box limits, first and third quartiles; whiskers, 1.5x interquartile range; black points, outliers.

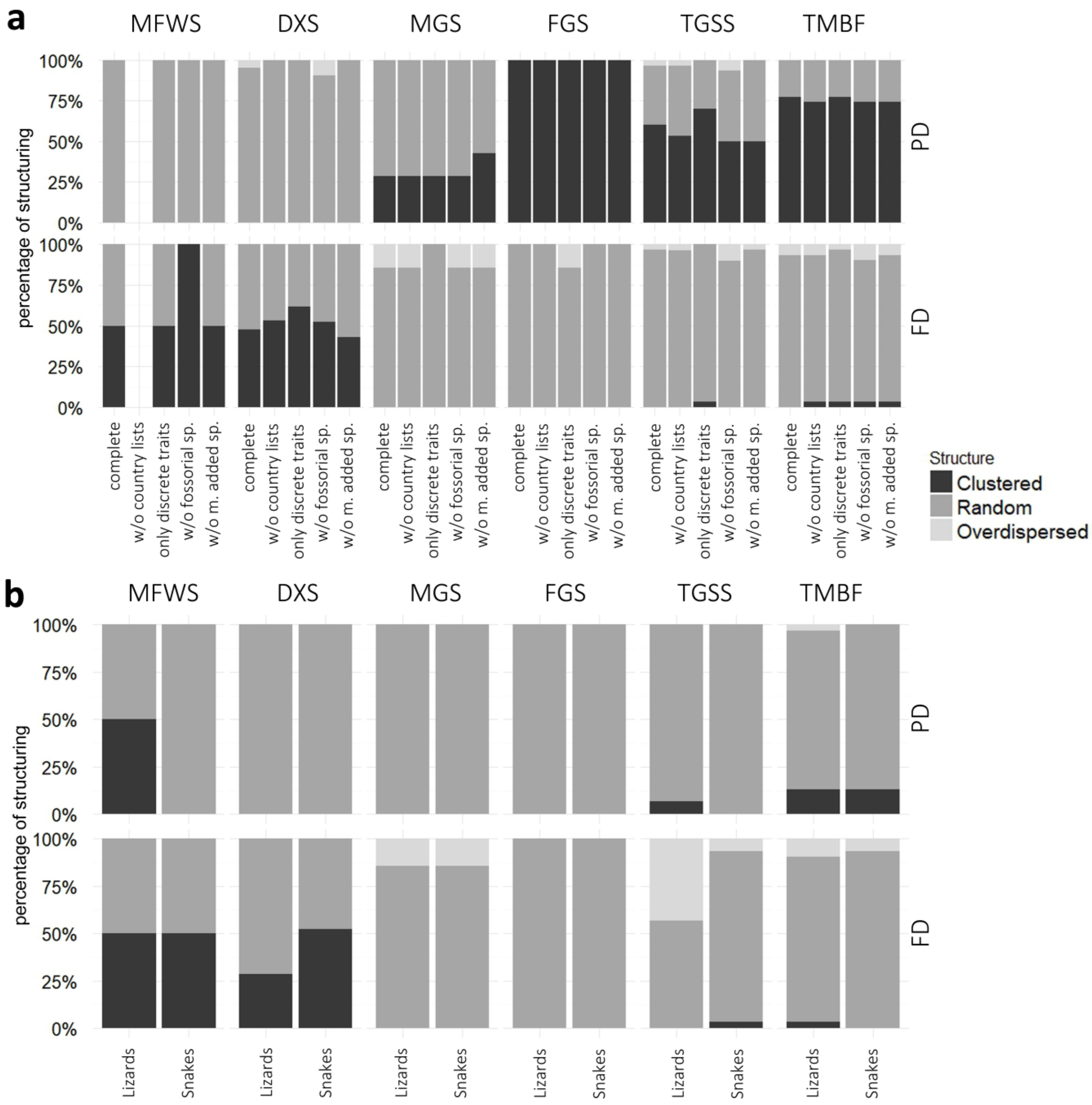

Supplementary Figure 4. NRI vs continental species pool. Proportions of significantly clustered, overdispersed and randomly structured local communities (NRI) in different biomes (95% CI, tested against the continental species pool), according to phylogenetic (PD) and functional (FD) diversity. **a** Complete dataset of all African squamates in comparison with different subsets (without country lists, only species with discrete lifestyle traits, without fossorial species, without manually added species), created to test data robustness. **b** Comparison of lizards and snakes. Abbreviations: MFWS (Mediterranean forests, woodlands & scrubs), DXS (deserts & xeric shrublands), MGS (montane grasslands & shrublands), FGS (flooded grasslands & savannahs), TGSS (tropical grasslands, savannahs & shrublands), TMBF (tropical moist broadleaf forests).

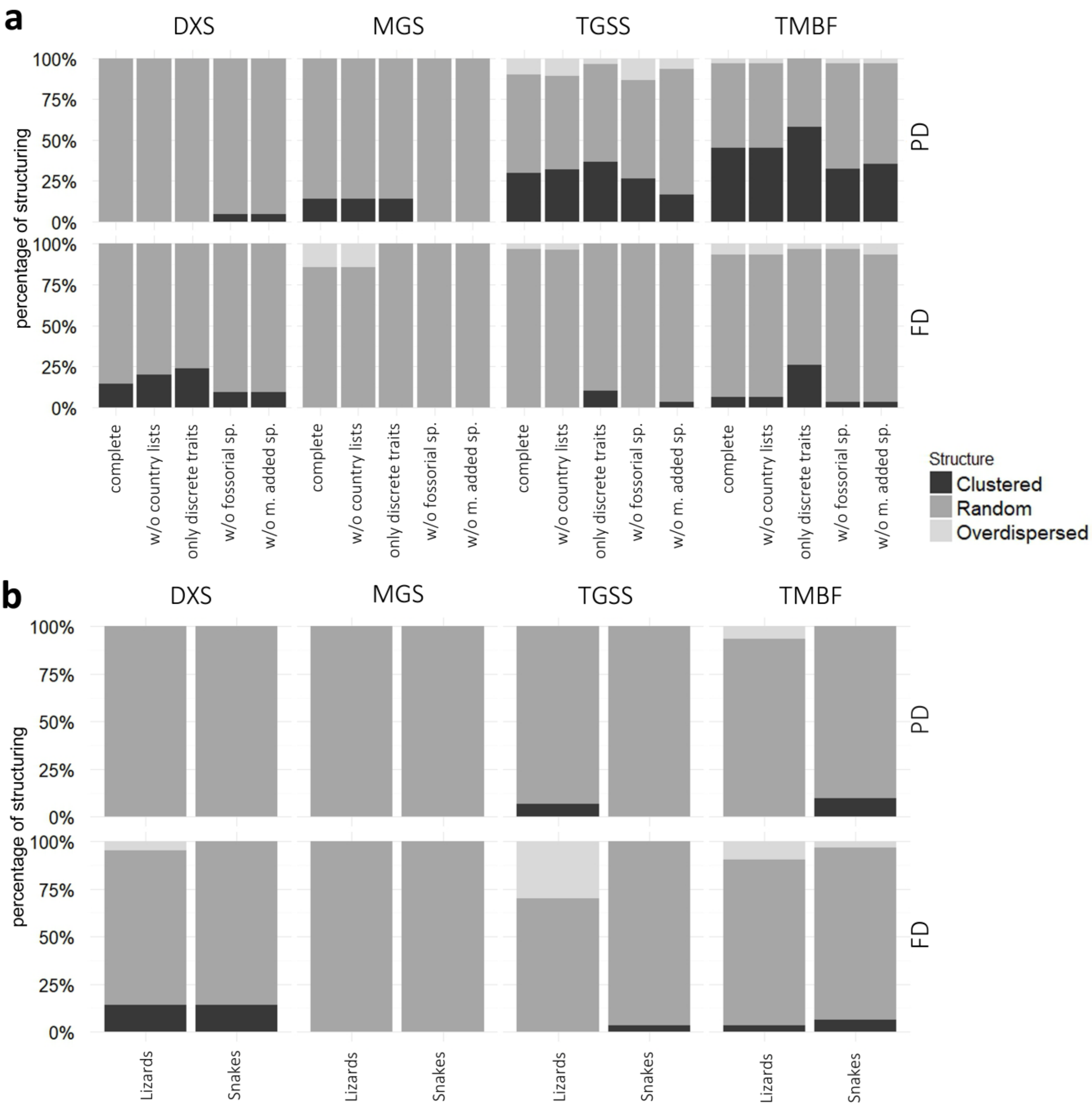

Supplementary Figure 5. NRI vs biome species pool. Proportions of significantly clustered, overdispersed and randomly structured local communities (NRI) in different biomes (95% CI, tested against the biome species pool), according to phylogenetic (PD) and functional (FD) diversity. **a** Complete dataset of all African squamates in comparison with different subsets (without country lists, only species with discrete lifestyle traits, without fossorial species, without manually added species), created to test data robustness. **b** Comparison of lizards and snakes. Abbreviations: DXS (deserts & xeric shrublands), MGS (montane grasslands & shrublands), TGSS (tropical grasslands, savannahs & shrublands), TMBF (tropical moist broadleaf forests).

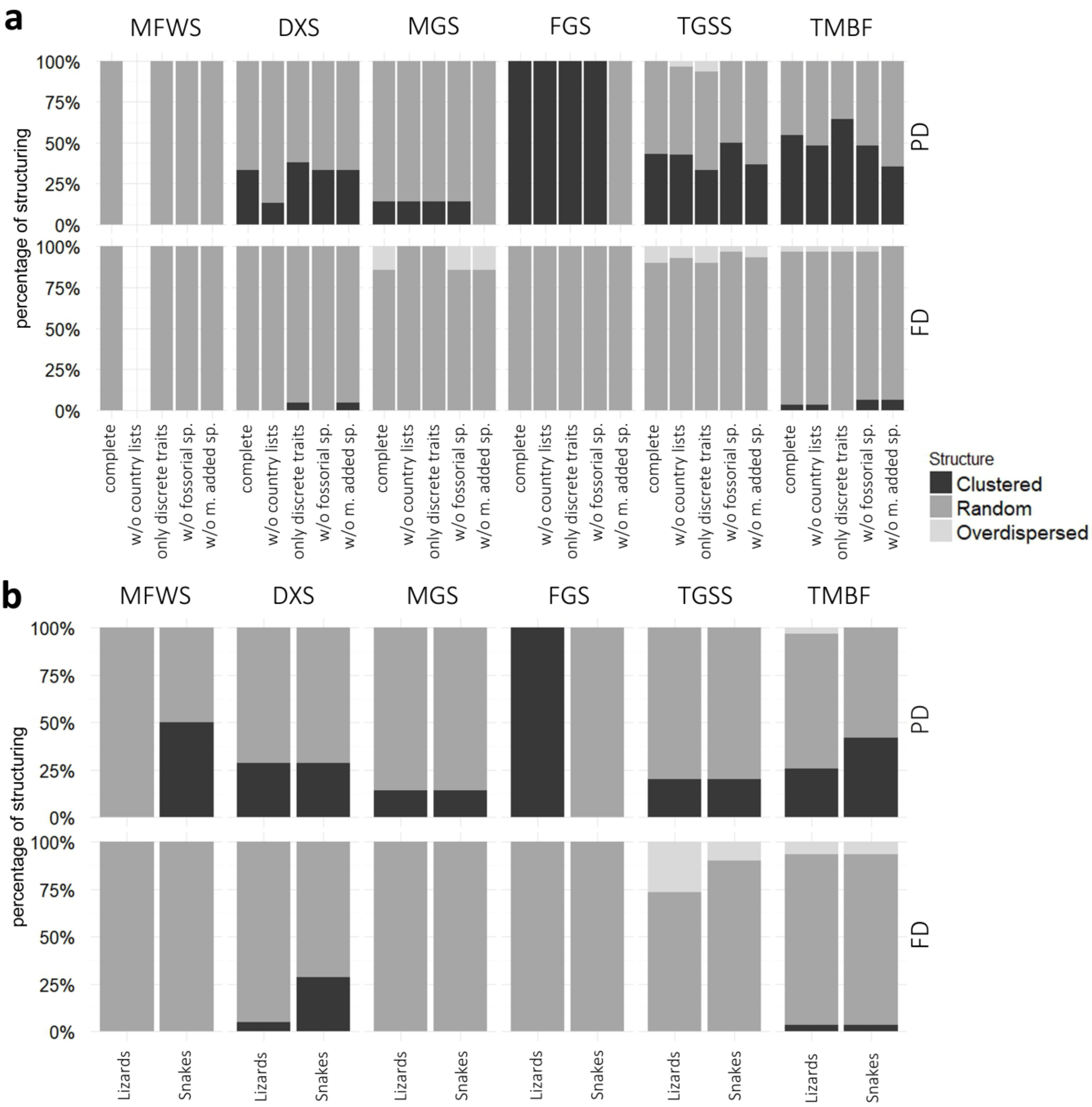

Supplementary Figure 6. NTI vs continental species pool. Proportions of significantly clustered, overdispersed and randomly structured local communities (NTI) in different biomes (95% CI, tested against the continental species pool), according to phylogenetic (PD) and functional (FD) diversity. **a** Complete dataset of all African squamates in comparison with different subsets (without country lists, only species with discrete lifestyle traits, without fossorial species, without manually added species), created to test data robustness. **b** Comparison of lizards and snakes. Abbreviations: MFWS (Mediterranean forests, woodlands & scrubs), DXS (deserts & xeric shrublands), MGS (montane grasslands & shrublands), FGS (flooded grasslands & savannahs), TGSS (tropical grasslands, savannahs & shrublands), TMBF (tropical moist broadleaf forests).

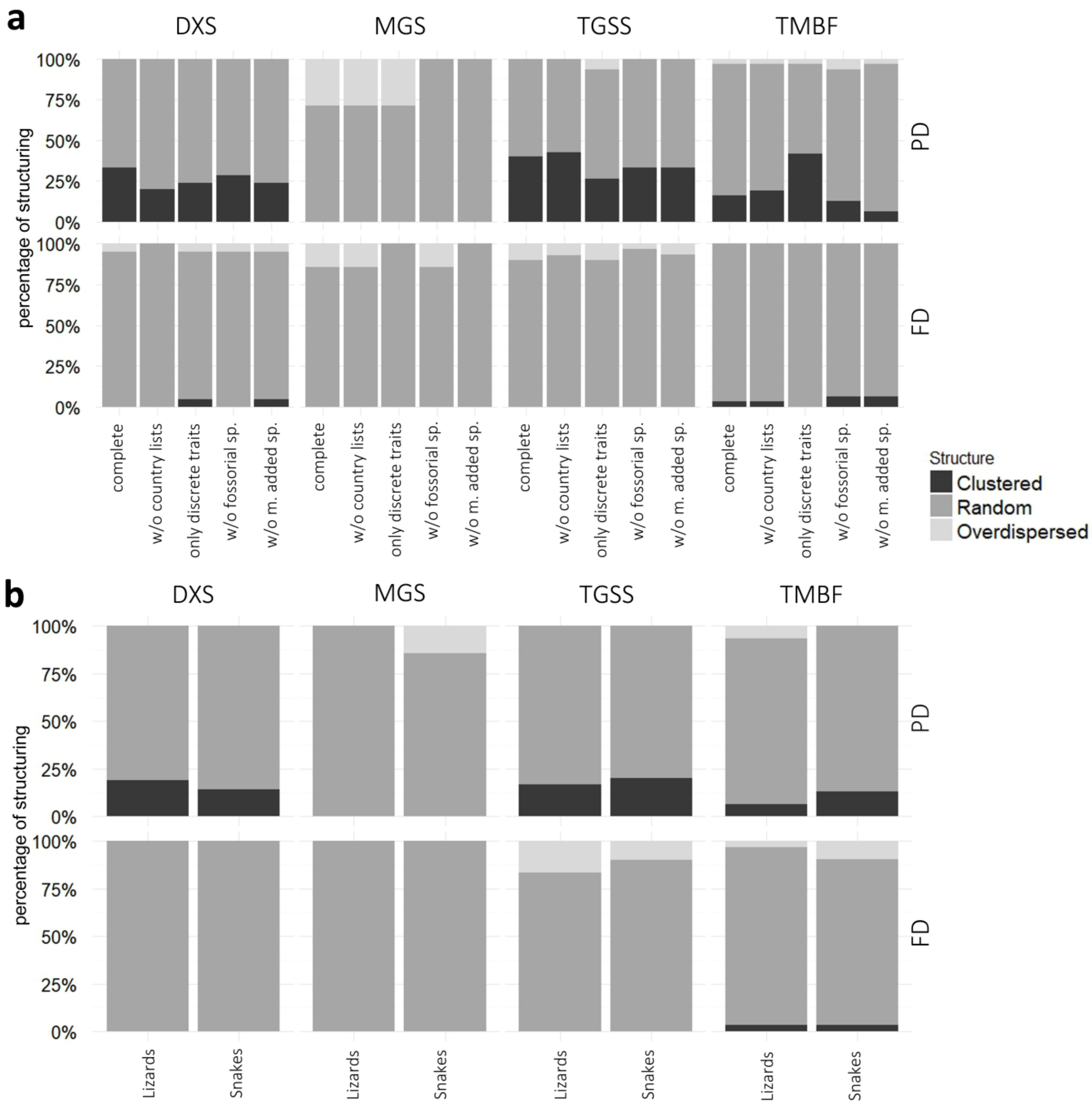

Supplementary Figure 7. NTI vs biome species pool. Proportions of significantly clustered, overdispersed and randomly structured local communities (NTI) in different biomes (95% CI, tested against the biome species pool), according to phylogenetic (PD) and functional (FD) diversity. **a** Complete dataset of all African squamates in comparison with different subsets (without country lists, only species with discrete lifestyle traits, without fossorial species, without manually added species), created to test data robustness. **b** Comparison of lizards and snakes. Abbreviations: DXS (deserts & xeric shrublands), MGS (montane grasslands & shrublands), TGSS (tropical grasslands, savannahs & shrublands), TMBF (tropical moist broadleaf forests).

Supplementary Table 1. Calibration points

Split	Min (mya)	Max (mya)	Used species	Source
Lepidosauria	238,00	249,60	*Sphenodon punctatus, Dibamus bourreti*	Jones et al. (2013)
Eublepharidae-Sphaerodactylidae	44,00	63,30	*Eublepharis macularius, Sphaerodactylus fantasticus*	Jones et al. (2013)
Xantusia-Cordylus	61,00	84,30	*Cricosaura typica, Cordylus tropidosternum*	Jones et al. (2013)
Lacertidae-Amphisbaenidae	61,00	80,90	*Psammodromus hispanicus, Amphisbaena brasiliana*	Jones et al. (2013)
Anguimorpha	148,00	156,80	*Elgaria kingii, Python regius*	Jones et al. (2013)
Varanus-Lanthanotus	48,00	58,30	*Lanthanotus borneensis, Varanus griseus*	Jones et al. (2013)
Heloderma-Aniella	98,00	108,00	*Heloderma suspectum, Aniella pulchra*	Jones et al. (2013)
Elgaria-Ophisaurus	33,00	42,10	*Elgaria coerulea, Ophisaurus ventralis*	Jones et al. (2013)
Chamaeleo-Calumma	19,00	29,60	*Calumma brevicorne, Chamaeleo rudis*	Jones et al. (2013)
Physignathus-Ctenophorus	16,00	26,90	*Physignathus cocincinus, Ctenophorus maculosus*	Jones et al. (2013)
Gambelia-Anolis	48,00	58,00	*Gambelia wislizenii, Anolis boettgeri*	Jones et al. (2013)
Shinisaurus-Elgaria	128,00	134,40	*Shinisaurus crocodilurus, Elgaria paucicarinata*	Jones et al. (2013)
Serpentes	98,32	113,00	*Liotyphlops albirostris, Natrix natrix*	Head (2015)
Caenophidia	66,00	72,10	*Python sebae, Acrochordus javanicus*	Head et al. (2016)

Supplementary References

Independent phylogenies used to insert species manually into the tree of Pyron et al. (2013)

1. Lenk, P., Herrmann, H.-W., Joger, U. & Wink, M. Phylogeny and taxonomic subdivision of *Bitis* (Reptilia: Viperidae) based on molecular evidence. *Darmstädter Beiträge zur Naturgeschichte* 8, 31–38 (1999).

2. Wilms, T.M., Böhme, W., Wagner, P., Lutzmann, N. & Chmitz, A. On the phylogeny and taxonomy of the genus *Uromastyx* Merrem, 1820 (Reptilia : Squamata : Agamidae : Uromastycinae) – resurrection of the genus *Saara* Gray, 1845. *Bonner zool. Beiträge* 56, 55–99 (2009).

3. Stanley, E.L., Bauer, A.M., Jackman, T.R., Branch, W.R. & Mouton, P.L.F.N. Between a rock and a hard polytomy: Rapid radiation in the rupicolous girdled lizards (Squamata: Cordylidae). *Mol. Phylogenet. Evol.* 58, 53–70 (2011).

4. Bates, M.F. et al. A molecular phylogeny of the African plated lizards, genus *Gerrhosaurus* Wiegmann, 1828 (Squamata: Gerrhosauridae), with the description of two new genera. *Zootaxa* 3750, 465–493 (2013).

5. Edwards, S. et al. Taxonomic adjustments in the systematics of the southern African lacertid lizards (Sauria: Lacertidae). *Zootaxa* 3669, 101–114 (2013).

6. Measey, G.J. & Tolley, K.A. A molecular phylogeny for sub-Saharan amphisbaenians. *Afr. J. Herpetol.* 62, 100–108 (2013).

7. Leaché, A.D. et al. A hybrid phylogenetic-phylogenomic approach for species tree estimation in African *Agama* lizards with applications to biogeography, character evolution, and diversification. *Mol. Phylogenet. Evol.* 79, 215–230 (2014).

Literature used for compiling the species composition data

Northern Marokko

8. Mediani, M., Brito, J.B. & Fahd, S. Atlas of the amphibians and reptiles of northern Morocco: updated distribution and patterns of habitat selection. *Basic Appl. Herpetol.* 29, 81-107 (2015).

Marokko

9. Harris, D.J., Perera, A., Bartara, M., Tarroso, P. & Salvi, D. New distribution notes for terrestrial herpetofauna from Morocco. *North-Western J. Zool*. 6, 309-315 (2010).

Southern Tunisia

10. Joger, U. Reptiles and amphibians of southern Tunisia. *Kaupia* 12, 71-88 (2003).

El Alamein

11. El Din, S.B. (2006). A Guide to the Reptiles and Amphibians of Egypt. Oxford University Press. Oxford, UK, 31-40

Lybia

12. Frynta, D., Kratochvíl, L., Moravec, J., Benda, P., Dandová, R., Kaftan, M., et al. Amphibians and reptiles recently recorded in Lybia. *Acta Soc. Zool. Bohem.* 64, 17-26 (2000).

Southern Lybia

13. Ibrahim, A.A. Contribution to the herpetology of southern Libya. *Acta Herpetol.* 3, 35-49 (2008).

Fezzan Province

14. Essighaier, M.F.A., Taboni, I.M., Etayeb, K.S. The diversity of wild animals at Fezzan Province (Lybia). *Biodivers. J.* 6, 245-252 (2015).

Western Sahara

15. Geniez, P., Mateo, J.-A., Bons, J. A checklist of the amphibians and reptiles of Western Sahara. *Herpetozoa* 13, 149-163 (2000).

Wadi El Gemal

16. El Din, S.B. (2006). A Guide to the Reptiles and Amphibians of Egypt. Oxford University Press, Oxford, UK, 31-40

Gebel Elba

17. El Din, S.B. (2006). A Guide to the Reptiles and Amphibians of Egypt. Oxford University Press, Oxford, UK, 31-40

Gebel Uweinat

18. El Din, S.B. (2006). A Guide to the Reptiles and Amphibians of Egypt. Oxford University Press, Oxford, UK, 31-40

Mauritania

19. Pardial, J.M. Commented distributional list of the reptiles of Mauritania (West Africa). *Graellsia* 62, 159-178 (2006).

Kamanjab

20. Bauer, A.M., Branch, W.R. & Haacke, W.D. The herpetofauna of the Kamanjab area and adjacent Damaraland, Namibia. *Madoqua* 18, 117-145 (1993).

Red Sea Hills

21. Müller, J., Hipsley, C, Kirchhof, S. & Mahmoud, Z. New investigations on the herpetofauna of the Red Sea Hills of Sudan, Preliminary Results of a field trip in October 2011. Poster, Museum für Naturkunde Berlin (2011).

Southern Niger

22. Gonçalves, D. V., Álvares, F. & Brito, J.C. Data on the distribution of herpetofauna of southern Niger with comments on Termit & Tin Toumma National Nature Reserve. *Bol. Asoc. Herpetol*. 24, 74-79 (2013).

Eritrea

23. Largen, M.J. An annotated checklist of the amphibians and reptiles of Eritrea, with keys for their identification. *Trop. Zool.* 10, 63-115 (1997).

South Eastern Senegal

24. Joger, U. & Lambert, M.R.K. Inventory of amphibians and reptiles in SE Senegal, including the Niokola-Koba National Park, with observations on factors influencing diversity. *Trop. Zool.* 15, 165-185 (2002).

W NP

25. Chirio, L. Inventaire des reptiles de la région de la Réserve de Biosphere Transfrontaliere du W (Niger/Bénin/Burkina Faso: Afrique de l'Ouest). *Bull. Soc. Herpétol. Fr.* 132, 13-41 (2009).

Djibouti

26. Ineich, I. Reptiles & amphibiens de la République de Djibouti. *Mission herpétologique* 1-60 (1999).

Sangaredi

27. Chirio, L. Inventaire des reptiles de la région de Sangarédi (Guinée maritime). *Bull. Soc. Herpétol. Fr.* 144, 67-100 (2012).

Upper Niger National Reserve

28. Greenbaum, E. & Carr, J. R. The herpetofauna of Upper Niger National Park, Guinea, *West Africa. Nat. Hist. Mus.* 37, 1-27 (2005).

Outambi & Kilimi NP

29. Zug, G.R. Amphibians and reptiles of the Outamba-Kilimi region, Sierra Leone. *J. Herpetol. Afr.* 33, 1-4 (1987).

Awash NP

30. Lanza, B. The reptiles of the awash national park (Shoa, Ethiopia). *Monit. Zool. Italia.* 4, 159-184 (1972).

Comoé NP

31. Rödel, M.-O. Die Echsen des Comoé-Nationalparks, Elfenbeinküste. *Salamandra* 33, 225-240 (1997).

32. Rödel, M.-O., Kouadio, K. & Mahsberg, D. Die Schlangenfauna des Comoé-Nationalparks, Elfenbeinküste: Ergänzungen und Ausblick. *Salamandra* 35, 165–180 (1999).

33. Hallermann, J. & Rödel, M.-O. A new species of Leptotyphlops (Serpentes: Leptotyphlopidae) of the longicaudus-group from West Africa. *Stuttg. Beitr. Naturk. A* 532, 1-8 (1995).

34. Rödel, M.-O., Grabow, K., Böckheler, C. & Mahsberg, D. Die Schlangen des Comoé-Nationalparks, Elfenbeinküste (Reptilia: Squamata: Serpentes). *Stuttg. Beitr. Naturk. A* 528, 1-18 (1995).

35. Rödel, M.-O. & Grabow, K. Zur Kenntnis von *Cynisca rouxae* (Hahn, 1979). *Salamandra* 32, 13–22 (1996).

Togo

36. Segniagbeto, G.H., Trape, J.F., David, P., Ohler, A., Dubois, A. & Adolé, G. The snake fauna of Togo: systematics, distribution and biogeography, with remarks on selected taxonomic problems. *Zoosystema* 33, 325-360 (2012).

37. Segniagbeto, G.H., J.-F. Trape, K.M. Afiademanyo, M.-O. Rödel, A. Ohler, A. Dubois, et al. Checklist of the lizards of Togo (West Africa), with comments on systematics, distribution, ecology, and conservation. *Zoosystema* 37, 381–402 (2015).

Ziama Forest

38. Böhme, W. (1994). Frösche und Skinke aus dem Regenwaldgebiet Südost-Guineas, Westafrika. II. Ranidae, Hyperoliidae, Scincidae; faunistisch-ökologische Bewertung. *Herpetofauna* 16, 6-16.

39. Böhme, W. Diversity of a snake community in a Guinean rain forest (Reptilia, Serpentes). In: *Isolated vertebrate communities in the tropics* (ed. Rheinwald, G.). Proc. 4th Int. Symp. Bonn. Bonn. zool. Monogr. 46, 69-78 (2000).

40. Böhme, W., Rödel, M.-O., Brede, C. & Wagner, P. The reptiles (Testudines, Squamata, Crocodylia) of the forested southeast of the Republic of Guinea (Guinée forestière), with a country-wide checklist. *Zool. Bull.* 60, 35-61 (2011).

Mount Nimba

41. Ineich, I. Contribution à la connaissance de la biodiversité des régions afro-montagnardes: les Reptiles du mont Nimba. Le peuplement animal du Mont Nimba (Guinée, Côte d'Ivoire, Liberia). *Mém. Mus. Nat. Hist. Nat.* 190, 597-637 (2003).

42. Rödel, M.-O. et al. including in this study

Sangba

43. Chirio, L. & Ineich, I. Biogeography of the reptiles of the Central African Republic. *Afr. J. Herpetol.* 55, 23-59 (2006).

Tchabal Mbabo Mountains

44. Herrmann, H.-W., Schmitz, A., Herrmann, P.A. & Böhme, W. Amphibians and reptiles of the Tchabal Mbabo mountains, Adamaoua plateau, Cameroon. *Bonner zool. Beitr.* 55, 27-35 (2007).

Togo Hills

45. Leaché, A.D., Rödel, M.-O., Linkem, C.W., Diaz, R.E., Hillers, A. & Fuijta, M.K. Biodiversity in a forest island: reptiles and amphibians of the West African Togo Hills. *Amphib. Reptile Conserv.* 4, 22-45 (2006).

Forêt de Lama

46. Ullenbruch, K. Untersuchungen zur Biodiversität der Amphibien und Reptilien eines isolierten Regenwaldes in der Dahomey Gap, Benin. Unpublished diploma thesis, Zoologisches Forschungsinstitut und Museum Alexander Koenig, Bonn (2003).

47. Ullenbruch, K., Grell, O. & Böhme, W. Reptiles from southern Benin, West Africa, with the description of a new Hemidactylus (Gekkonidae), and a country-wide checklist. *Bonn zool. Bull.* 57, 31-54 (2010).

Lamto

48. Roux-Esteve, R. Les serpents de la région de Lamto (Côte d'Ivoire). *Ann. Univ. Abidjan, sér. E* 2, 81-140 (1969).

49. Barbault, R. Les peuplements de lézards des savanes de Lamoto (Côte d'Ivoire). *Ann. Univ. Abidjan, sér. E* 8, 147-221 (1975).

50. Rödel, M.-O. Die Echsen des Comoé-Nationalparks, Elfenbeinküste. *Salamandra* 33, 225-240 (1997).

Mount Oku

51. Ineich, I., leBreton, M., Lhermitte-Vallarino, N. & Chirio, L. The reptiles of the summits of Mont Oku and the Bamenda Highlands, Cameroon. *Amphi. Rept. Conserv.* 9, e108 (2015).

Takamanda Forest Reserve

52. LeBreton, M., Laurent, C. & Foguekem, T. Reptiles of Takamanda Forest Reserve, Cameroon. *SI/MAB Series* 8, 83-94 (2003).

Seko

53. Chirio, L. & Ineich, I. Biogeography of the reptiles of the Central African Republic. *Afr. J. Herpetol.* 55, 23-59 (2006).

Mawne Forest Reserve

54. Foguekem, D. & LeBreton, M. Reptiles of Mawne Forest Reserve, Cameroon: with notes on the status, ecology and conservation of species. CAMHERP project. Cameroon Biodiversity Conservation

Society. Prepared for the Wildlife Conservation Society and GTZ (2002).

Tai NP

55. Rödel, M.-O. including in this study

56. Rödel, M.-O. & Mahsberg, D. Vorlaufige Liste der Schlangen des Tai-Nationalparks/Elfenbeinkuste und angrenzender Gebiete. *Salamandra* 36, 25-38 (2000).

57. Ernst, R. & Rödel, M.-O. A new Atheris species (Serpentes: Viperidae), from Taï National Park, Ivory Coast. *Herpetol. J.* 12, 55–61 (2002).

Mboki

58. Chirio, L. & Ineich, I. Biogeography of the reptiles of the Central African Republic. *Afr. J. Herpetol.* 55, 23-59 (2006).

Korup NP

59. Lawson, D.P. (1993). The reptiles and amphibians of the Korup National Park project, Cameroon. *Herpet. Nat. Hist.* 1, 27-90.

Mount Nlonako

60. Herrmann, H.-W., Böhme, W., Euskirchen, O., Herrmann, P.A. & Schmitz, A. African biodiversity hotspots: the reptiles of Mt Nlonako, Cameroon. *Rev. Suisse Zool.* 112, 1045-1069 (2005).

Ouazona

61. Chirio, L. & Ineich, I. Biogeography of the reptiles of the Central African Republic. *Afr. J. Herpetol.* 55, 23-59 (2006).

Zimba

62. Chirio, L. & Ineich, I. Biogeography of the reptiles of the Central African Republic. *Afr. J. Herpetol.* 55, 23-59 (2006).

Mount Cameroon

63. Legrand, G.N. Reptiles of mount Cameroon with specific reference to species in intercontinental trade. M. Sc. Thesis, University of Yaounde, Yaounde, Cameroon (2011).

Garamba NP

64. Dewitte, G.F. Reptiles. Exploration du Parc National de la Garamba, Mission H. De Saeger (1949-1952). Fascicle 48, Institut des Parcs Nationaux du Congo Belge, Bruxelles (1966).

S.C.A.D

65. Chirio, L. & Ineich, I. Biogeography of the reptiles of the Central African Republic. *Afr. J. Herpetol.* 55, 23-59 (2006).

Turkana

66. Zilliani, U., Sindaco, R., Razzetti, E., Wasonga, V., Modrý, D., Necas, P., et al. The herpetofauna of the eastern side of the Lake Turkana (Northern Kenya). *Riassunti del 6° Congresso Nazionale della Societas Herpetologica Italica*, 192-193 (2006).

Monts de Crystal

67. Pauwels, O., Kamdem A.T. & Chimsunchart, C. Recherches sur l'herpétofaune du Massif du Chaillu, Gabon. *Bull. Roy. Sci. Nat. Bel.* 72, 47-57 (2002).

Kibale Forest

68. Vonesh, J. Natural history and biogeography of the amphibians and reptiles of Kibale National Park, Uganda. *Contemporary Herpetology* 4, 1-14 (2001).

Kakamega Forest

69. Wagner, P. & Böhme, W. Herpetofauna Kakamegensis–the amphibians and reptiles of Kakamega Forest, western Kenya. *Bonner zool. Beitr.* 55, 123-150 (2007).

Virunga NP

70. Dewitte, G.F. Batraciens et Reptiles. Exploration du Parc National Albert, Mission G. F. DeWitte (1933-1935). Fascicle 33, Institut des Parcs Nationaux du Congo Belge, Bruxelles (1941)

Lopé NP

71. Pauwels, O., Christy, P. & Honorez, A. Reptiles and national parks in Gabon, western central Africa. *Hamadryad* 30, 181 (2006).

Bwindi Impenetrable Forest

72. Drewes, R.C. & Vindum, J.V. Amphibians of the Impenetrable Forest, southwest Uganda. *J. Zool.* 108, 55–70 (1991).

73. Drewes, R.C. & Vindum, J.V. Amphibians and reptiles of the Bwindi-Impenetrable National Park. Unpublished report, Uganda Wildlife Authority, Kampala (1997).

Parc National de Volcans

74. Roelke, C.E. & Smith, E.N. Herpetofauna, Parc National des Volcans, North Province, Republic of Rwanda. *J. Spec. Lists Dist.* 6, 525-531 (2010).

Tana River

75. Malonza, P.K., Wasonga, V.D., Muchai, V., Rotich, D., Bwong, A. & Bauer, A.M. Diversity and biogeography of herpetofauna of the Tana River Primate National Reserve, Kenya. *J. East Afr. Nat. Hist.* 95, 95-109 (2016).

Loango

76. Pauwels, O., Christy, P. & Honorez, A. Reptiles and national parks in Gabon, western central Africa. *Hamadryad* 30, 181 (2006).

Monts Doudou

77. Burger, M., Branch, W.R. & Channing, A. Amphibians and reptiles of Monts Doudou, Gabon: species turnover along an elevational gradient. *California Acad. Sci. Mem.* 28, 145-186 (2004).

Moukalaba NP

78. Pauwels, O., Christy, P. & Honorez, A. Reptiles and national parks in Gabon, western central Africa. *Hamadryad* 30, 181 (2006).

Massif de Chaillu

79. Pauwels, O., Kamdem A.T. & Chimsunchart, C. Recherches sur l’herpétofaune du Massif du Chaillu, Gabon. *Bull. Roy. Sci. Nat. Bel.* 72, 47-57 (2002).

Arusha NP

80. Razzetti, E. & Msuya, C.A. *Field guide to the amphibians and reptiles of Arusha National Park (Tanzania)*. (Istituto Oikos - TANAPA, Varese, 84 pp., 2002).

Arabuko Sokoke Forest

81. KIFCON (Kenyan indigenous forest conservation project). *Arabuko Sokoke Forest and Mida Creek: The official guide.* (Majestic Printing Works Ltd, Nairobi, Kenya, 1995).

Kitobo Forest

82. Malonza, P.K., Beryl A.B. & Muchai, A. Kitobo Forest of Kenya, a unique hotspot of herpetofaunal diversity. *Acta. Herpetol.* 6, 149-160 (2011).

Usambara Mountains

83. Howell, K.M. Herpetofauna of the East African forests. In: *Biogeography and ecology of the rain forests of Eastern Africa.* (eds. Lovett, J.C. & S. Wasser). Cambridge University Press, Cambridge, pp. 173-201 (1993).

Katavi NP

84. Caro, T., Ewans, O.S., Fitzherbert, E., Gardner, E.A., Howell, K., Drewes, R., et al. Reptiles of Katavi National Park, western Tanzania, are from different biomes. *Afr. J. Ecol.* 49, 377-382 (2011).

Udzungwe Mountains

85. Howell, K.M. Herpetofauna of the East African forests. In: *Biogeography and ecology of the rain forests of Eastern Africa.* (eds. Lovett, J.C. & S. Wasser). Cambridge University Press, Cambridge, pp. 173-201 (1993).

Katanga

86. Broadley, D.G. & Cotterill, D.P.F. The reptiles of southeast Katanga, an overlooked 'hot spot'. *Afr. J. Herpetol.* 53, 35-61 (2004).

Capanda Dam Region

87. Ceríaco, L.M., Bauer, A.M., Blackburn, D.C. & Lavres A.C.F. The Herpetofauna of the Capanda Dam Region, Malanje, Angola. *Herpetol. Rev.* 45, 667-674 (2014).

Cangalada

88. Pires Ceríaco, L.M., Pimentel Marques, M. & Bandeira, S.A. Anfíbios e réptais do Parque Nacional da Cangandala. *Insituto Nacional da Biodiversidade e Áraes de Concervacao & Museo Nacional de Historia Natural e da Ciência* (2016).

Nyika Plateau

89. Stewart, M.M. & Wilson, V.J. Herpetofauna of the Nyika Plateau (Malawi and Zambia). *Annals. Nat. Mus.* 18, 287-313 (1966).

Niassa Game Reserve

90. Branch, W.R., Rödel, M.-O. & Marais, J. Herpetological survey of the Niassa Game Reserve, northern Mozambique-Part I: Reptiles. *Salamandra* 42, 195 (2005).

91. Branch, W.R., Rödel. M.-O. & Marais, J. A new species of rupicolous Cordylus Laurenti 1768 (Sauria: Cordylidae) from Northern Mozambique. *Afr. J. Herpetol.* 54, 131–138 (2005).

Luangwa Valley

92. Wagner, P. including in this study

Kasanka NP

93. Homepage of the Kasanka National Park (2017). Available at: http://www.kasanka.com/habitats_and_wildlife.htm

Mulanje Mountain

94. Broadley, D.G. An annotated check list of the herpetofauna of Mulanje Mountain. *Nyala* 21, 29-36 (2001).

Four Corner NP

95. Timberlake, J.R. & Childes, S.L. Biodiversity of the Four Corners Area: Technical Reviews Volume Two (Chapters 5-15). *Occ. Public. Biodivers.* 15, 21-30 (2004).

Gorongosa

96. Rödel, M.-O. et al. including in this study

Matobo

97. Broadley, D.G. & Wilson, V.J. The reptiles and amphibians of the Matobo Hills, Zimbabwe. *Arnoldia Zimbabwe* 10, 309-339 (2011).

Brandberg

98. Van den Elzen, P. The herpetofauna of Brandberg, Southwest Africa. *Bonn. zool. Beitr.* 34, 293-309 (1983).

Parque Nacional de Banhine

99. Pietersen, D.W., Pietersen, E.W. & Haacke, W.D. First herpetological appraisal of the Parque Nacional de Banhine, Gaza Province, southern Mozambique. *Annls. Dits. Nat. Mus. Nat. Hist*. 3, 153–163 (2013).

Langjan Nature Reserve

100. Schmidt, A.D. The herpetofauna of the Langjan Nature Reserve (Limpopo Province, Republic of South Africa). *Herpetozoa* 15, 121-135 (2002).

Soutpansberg

101. Kirchhof, S., Krämer, M., Linden, J. & Richter, K. The reptile species assemblage of the Soutpansberg (Limpopo Province, South Africa) and its characteristics. *Salamandra* 46, 147-166 (2010).

Blouberg Nature Reserve and adjacent areas

102. Schmidt, A.D., Snyman, P.H. & Gruschwitz, M. Diversity of reptiles in the bushveld of the Blouberg Nature Reserve (Limpopo Province, South Africa). *Herpetozoa* 18, 35-53 (2005).

Krueger NP

103. Anonymous. Checklist of the reptiles of the Krueger National Park (1991).

Lobatsi-Linokana Area

104. Power, J.H. On the herpetological fauna of the Lobatsi-Linokana area. *Trans. Roy. Soc. South Afr.* 14, 405-422 (1926).

Ndumu Game Reserve

105. Pooley, T. A preliminary checklist of the reptiles found within the Ndumu and Mkuzi Game Reserves in northern Zululand. *Lammergeyer* 11, 65-67 (1965).

Sperrgebiet

106. Branch, W.R. Herpetofauna of the Sperrgebeit region of southern Namibia. *Herpetol. Nat. Hist.* 2, 1-11 (1994).

Sodwana Bay

107. Haagner, G.V. (1994). A checklist and biogeographic appraisal of the herpetofauna of the Sodwana Bay National Park and adjacent lakes area. *Durban Mus. Nov.* 19, 30-40 (1994).

Mkuzi Game Reserve

108. Pooley, T. A preliminary checklist of the reptiles found within the Ndumu and Mkuzi Game Reserves in northern Zululand. *Lammergeyer* 11, 65-67 (1965).

Richtersveld NP

109. Bauer, A.M. & Branch, W.R. The herpetofauna of the Richtersveld National Park and the adjacent northern Richtersveld, Northern Cape Province, Republic of South Africa. *Herpetol. Nat. Hist.* 8, 111-160 (2001).

Oribi Gorge Nature Reserve

110. Bourquin, O. & Mathias, I. The vertebrates of Oribi Gorge Nature Reserve: 1. *Lammergeyer* 33, 35-44 (1984).

Nieuwoudtville Area

111. Mouton, P.F.N. & Alblas, A. Conservation Farming Project, Reptile Diversity in the Nieuwoudtville Area. *National Botanical Institute* (2002).

112. Branch, W.R. *Field guide to Snakes and other Reptiles of Southern Africa. 3rd Edition*. (Struik, Cape Town , 1998).

Karoo NP

113. Branch, W.R. & Braack, H.H. Reptiles and amphibians of the Karoo national park. A surprising diversity. *J. Herpetol. Ass. Afr.* 36, 26-35 (1989).

Cape Province

114. Branch, W.R. The herpetofauna of the Cape Province, South Africa: new distribution records and zoogeography. *J. Herpetol. Ass. Afr.* 37, 17-44 (1990).

Tsitsikamma Forest

115. Branch, W.R. & Hanekom, N. The herpetofauna of the Tsitsikamma coastal and forest national parks. *Koedoe* 30, 49-60 (1987).

Literature used for compiling the lifestyle trait data

116. Bates, M.F., Tolley, K.A., Edwards, S., Davids, Z., Da Silva, J.M. & Branch, W.R. A molecular phylogeny of the African plated lizards, genus Gerrhosaurus Wiegmann, 1828 (Squamata: Gerrhosauridae), with the description of two new genera. *Zootaxa* 3750, 465-493 (2013).

117. Branch, W. *Field Guide to Snakes and Other Reptiles of Southern Africa. 3. Edition.* (Ralph Curtis Books Sanibel Island, FL, 1998).

118. Harris, D.J. & Arnold, E.N. Elucidation of the relationships of spiny-footed lizards, Acanthodactylus spp. (Reptilia: Lacertidae) using mitochondrial DNA sequence, with comments on their biogeography and evolution. *J. Zool.* 252, 351–365 (2000).

119. Leaché, A.D.L., Chong, R.A., Papenfuss, T.J., Wagner, P., Böhme, W., Schmitz, A. et al. Phylogeny of the genus Agama based on mitochondrial DNA sequence data. *Bonner zool. Beiträge* 56, 273–278 (2009).

120. Lenk, P., Herrmann, H.-W., Joger, U. & Wink, M. Phylogeny and taxonomic subdivision of Bitis (Reptilia: Viperidae) based on molecular evidence. *Kaupia* 8, 31–38 (1999).

121. O´Shea, M. *Boas and Pythons of the World.* (New Holland & Princeton University Press, Princeton, NJ, 2007).

122. Sheehy III, C. *On The Structure And Function Of Tails In Snakes: Relative Length And Arboreality.* Master Thesis, University of Florida (2006).

123. Shine, R., Branch, W.R., Webb, J.K., Harlow, P.S., Shine, T., Shine, R., et al. Sexual dimorphism, reproductive biology, and dietary habits of psammophiine snakes (Colubridae) from Southern Africa. *Copeia* 2006, 650–664 (2006).

124. Spawls, S., Howell, K.M., Drewes R.C. & Ashe, J. A. *Field Guide to the Reptiles of East Africa.* (Bloomsbury Publishing, London, 2004).

125. Stanley, E.L., Bauer, A.M., Jackman, T.R., Branch, W.R. & Mouton, P.L.F.N. Between a rock and a hard polytomy: Rapid radiation in the rupicolous girdled lizards (Squamata: Cordylidae). *Mol. Phylogenet. Evol.* 58, 53–70 (2011).

126. The IUCN Red List of Threatened Species (2010). Available at: http://www.iucnredlist.org/. Last accessed 05.06.2017

127. Tolley, K. & Herrel, A. *The Biology of Chameleons*. (University of California Press, Berkeley and Los Angeles, CA, 2014).

128. Uetz, P., Freed, P. & Hošek, J. (2017). The Reptile Database. Available at: http://reptile-database.reptarium.cz/. Last accessed 15.06.2017

129. Vitt, L.J. & Caldwell, J.P. *Herpetology: An Introductory Biology of Amphibians and Reptiles. 4. Edition.* (Elsevier Ltd., Oxford, 2013).
